# Transcriptome-Wide Analysis of Brain Cancer Initiated by Polarity Disruption in *Drosophila* Type II Neuroblasts

**DOI:** 10.3390/ijms26115115

**Published:** 2025-05-26

**Authors:** Simona Paglia, Patrizia Morciano, Dario de Biase, Federico Manuel Giorgi, Annalisa Pession, Daniela Grifoni

**Affiliations:** 1 Department of “Pharmacy and Biotechnology”, University of Bologna, Via Selmi 3, 40126 Bologna, Italy; simona.paglia@ior.it (S.P.); dario.debiase@unibo.it (D.d.B.); federico.giorgi@unibo.it (F.M.G.); 2 Department of “Life Health and Environmental Sciences”, University of L’Aquila, Via Vetoio, Coppito 2, 67100 L’Aquila, Italy; patrizia.morciano@guest.univaq.it; 3 INFN-Laboratori Nazionali del Gran Sasso, 67100 Assergi, Italy; 4 Solid Tumor Molecular Pathology Laboratory, IRCCS Azienda Ospedaliero-Universitaria di Bologna, Via Massarenti 9, 40138 Bologna, Italy

**Keywords:** brain cancer, GBM, *Drosophila* model, polarity loss, type II neuroblasts, RNA-seq analysis

## Abstract

Brain tumors, in particular gliomas and glioblastoma multiforme (GBM), are thought to originate from different cells facing specific founding insults, a feature that partly justifies the complexity and heterogeneity of these severe forms of cancer. However, gliomas and GBM are usually reproduced in animal models by inducing molecular alterations in mature glial cells, which, though being part of the puzzle, do not represent the whole picture. To fill this conceptual gap, we previously developed a neurogenic model of brain cancer in *Drosophila*, demonstrating that the loss of cell polarity in neural stem cells (called neuroblasts in the fruit fly) is sufficient to promote the formation of malignant masses that continue to grow in the adult, displaying several phenotypic traits typical of human GBM. Here, we expand on previous work by restricting polarity disruption to *Drosophila* type II neuroblasts, whose self-renewal is comparable to that of mammalian neural progenitors, with the aim to capture the molecular signature of the resulting cancers in a specific and reproducible context. A comparison of the most deregulated transcripts with those found in human primary GBMs confirmed that our model can be proficiently used to delve into the roots of human brain tumorigenesis.

## 1. Introduction

Glioblastoma multiforme (GBM) is the most common and aggressive among adult brain tumors, and its prognosis, despite all the advances made in chemo- and radiotherapy, still remains poor [1]. One of the characteristics that makes GBM such an aggressive disease is, undoubtedly, its intrinsic phenotypic heterogeneity [2] and the presence of a highly undifferentiated cell population called “Glioblastoma Stem Cells” (GSCs), also identified as “Glioblastoma Tumor-Initiating Cells” (GTICs), which are responsible for tumor initiation [3], maintenance, and relapse [4]. The origin of GSCs is still controversial. In the past, the “no new neuron” dogma has imposed mature cells as the only possible origin, with these cells, following a process of dedifferentiation, acquiring some stem properties [5]. This stereotype changed with the discovery of adult neurogenesis. Scientists began wondering if GSCs could originate from normal neural stem cells (NSCs) which, due to the accumulation of mutations, were transformed into cancer stem cells [6], while other lines of research identified the cells of origin of GBM in committed progenitors, such as the Oligodendrocyte Progenitor Cells (OPCs) [7]. The mechanisms involved are possibly manifold, and the hypotheses are not mutually exclusive, given the complex and chaotic nature of cancer. However, it is more likely that mutations are fixed in proliferating stem cells rather than in post-mitotic cells, as oncogenic mutations are rare and stochastic events. Thinking in these terms, glioblastoma is a “bad luck cancer”, whose incidence is strongly correlated with the total number of cells and divisions of the stem compartment [8]. Recent work has also shown that adult neural populations in progressive stages of differentiation show decreasing susceptibility to malignant transformation [9]. In the effort to unravel such a tangled disease, the use of animal models is particularly relevant, as in vivo studies allow the drawback of in vitro models to be overcome, as these fail to recreate the complex environment of the nervous system and its crosstalk with the tumor, essential to the proliferation and migration of tumor cells [10]. Besides the copious mouse models and despite the obvious anatomical differences, GBM is also modeled in the fruit fly [11]. Since activating mutations in the *Epidermal Growth Factor Receptor* (*EGFR*) and the inactivation of the *Phosphatase and TENsin homolog* (*PTEN*) are recurrent in *de novo* GBM [12], the most used model showed the co-activation of EGFR and PI3K (PTEN antagonist) in glial cells, resulting in the formation of neoplastic tumors with cells acquiring the ability to proliferate and invade [13]. This model has been and is still largely used to investigate the most disparate genetic and molecular aspects of gliomagenesis, for example, the unexpected relationship between glioma and neurodegeneration [14] or the role of autophagy in this pathology [15], but also to identify potential pharmacological targets [16]. However, although widely used, this fly model “starting” from mature glia cannot describe the entire disease, and our intent was to build a neurogenic model instead, to scrutinize tumorigenesis starting from neural stem cells. One of the mechanisms responsible for the maintenance of GSCs was recently identified in the PTEN/aPKC/Lgl molecular axis [17]. The most common lesion of primary GBMs is indeed the inactivation of *PTEN* [18]*,* a tumor suppressor gene (TSG) that negatively regulates the PI3K/AKT pathway [19]. When *PTEN* is lost, insulin signaling is constitutively active, and aPKC is activated [20]. aPKC is a kinase involved in the control of cell polarity regulation by phosphorylating different targets, including Lgl, which undergoes autoinhibition following phosphorylation [21]. *lgl* (*lethal giant larvae*) was the first TSG identified in *Drosophila* [22], and its product is a membrane protein involved in the maintenance of apical-basal polarity in epithelial cells [23] and neuroblasts (NBs) [24]. *Drosophila lgl* is evolutionarily conserved [25] and it is deregulated in different human cancers [26,27,28,29], stressing the link between polarity loss and cell proliferation control [30]. The expression of a non-phosphorylatable form of Lgl in human GBM cell lines reduces their motility in vitro and impairs invasion in mouse brains, as well as inducing GBM cell differentiation both in vitro and in vivo [31]. Given our expertise and our interest in the relationship between cell polarity and cancer, our aim was to evaluate the impact of polarity disruption in neural stem cells and its contribution to the tumorigenic process. In a previous work, we demonstrated that the PTEN/aPKC/Lgl axis is conserved in *Drosophila*, and that its alteration, with the following misregulation of cell polarity, causes the formation of brain tumors due to the accumulation of highly proliferating NBs. These masses persisted in the adult stage and continued to grow, leading the animals to premature death [32]. During the molecular characterization of the tumors, we noted that these masses also expressed PntP1, a specific marker of the type II subclass of neuroblasts [33], which consist of only eight cells per brain hemisphere, give rise to most neural cells of the adult CNS [34], and seem to be particularly prone to initiate malignant transformation [35]. Their lineage is similar to that of mammalian neural stem cells, as they originate transit-amplifying cells, called Intermediate Neural Progenitors (INPs), to expand the progenitor pool [36]. To understand whether this population may initiate neoplastic growth following polarity loss, we altered the PTEN/aPKC/Lgl axis specifically in type II NBs, and our results suggest that this is sufficient to initiate and support brain cancer in a reproducible manner. The purpose of this work was to refine our tumor model, so that it might be proficiently interrogated to obtain reliable and translatable molecular information on human brain tumorigenesis.

## 2. Results

### 2.1. The Alteration in Cell Polarity in Type II Neuroblasts Leads to the Formation of Adult Brain Tumors

**Figure 1:**
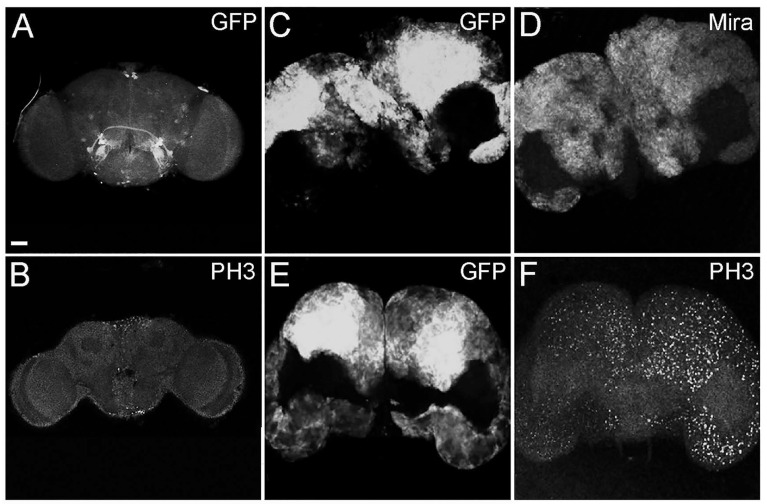
Representative images of *insc-GAL4, ase-GAL80-GFP-aPKC^CAAX-wt^* adult brains with GFP^+^ tumor masses (**C**,**E**) compared with a control *insc-GAL4, ase-GAL80-GFP* adult brain (**A**). Neural stem cells stained for Miranda (Mira) (**D**), and mitotic cells in control (**B**) and tumor brains (**F**) stained for PH3. All the images are at the same magnification (400×), and the scale bar (indicated in **A**) is 50 µm.

With the aim of altering polarity in type II NBs, we overexpressed the activated form of aPKC (aPKC^CAAX^) in this stem cell subpopulation. To specifically target type II NBs, we used a promoter obtained by the combined use of *inscuteable (ins)-Gal4* and *asense (ase)-Gal80*. *insc-GAL4* is active in all NBs, and *ase-GAL80* inhibits its expression in type I NBs; in this way, driver activity is restricted to type II NBs [37]. We also expressed UAS-GFP to label the sub-territory of this region (Figure 1A). The experimental progeny reached the adult stage, satisfying a pre-requisite of the model. Following aPKC activation, the adult brains appeared extremely enlarged, with an aberrant structure, and were characterized by the presence of large GFP-positive regions that filled about the 70% of the brain, calculated by averaging the GFP-positive areas of 100 adult tumor brains (Figure 1C,E).

In order to characterize these tumoral masses, we performed immunofluorescence analyses. In Figure 1, the adult brains were stained for Miranda (Mira), a marker specific to neural stem cells [38], and it can be appreciated that the GFP-labeled tumor areas were diffusely positive to Miranda staining (D). In the lower part of the panel, staining for Phospho-Histone H3 is shown in control (B) and tumor (F) brains. Histone H3 is specifically phosphorylated during cell division; in fact, it is used as a marker of mitosis [39]. These tumors are, therefore, characterized by being composed of neural stem cells that persist and continue dividing in the adult organ, contrary to what happens in wild type organs [40]. Being Mira and PH3 a lineage marker and a mitotic marker, respectively, their expression was intrinsically associated with tumor cells and covered the entire GFP-positive areas in all the analyzed samples.

### 2.2. Tumor Masses Cause Neuromotor Deficits, Leading Animals to Premature Death

**Figure 2:**
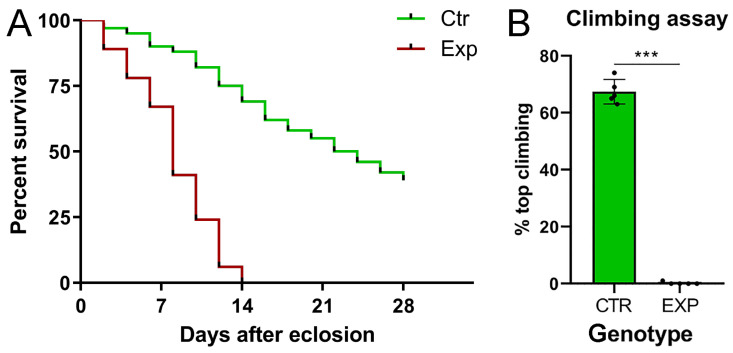
Kaplan–Meier survival curve and climbing assay of *insc-Gal4*, *ase-Gal80-GFP-aPKC^CAAX-wt^* adult flies. (**A**) The graph shows the survival percentage of *insc-Gal4, ase-Gal80-GFP* (CTR, green line) and *insc-Gal4*, *ase-Gal80-GFP-aPKC^CAAX-wt^* (EXP, red line) sibling flies counted every two days from eclosion to death. The *p*-value associated with the chi-square test proves that the difference is statistically significant (*p* ≤ 0.001). On the right, the climbing assay in *insc-Gal4*, *ase-Gal80-GFP-aPKC^CAAX-wt^* vs. *insc-Gal4, ase-Gal80-GFP* adult flies. (**B**) The graph shows the percentages of *insc-Gal4, ase-Gal80-GFP-aPKC^CAAX-wt^* (EXP) and *insc-Gal4*, *ase-Gal80-GFP* (CTR, green) individuals that reached the top line. Each dot in (**B**) represents a group of 100 flies, and error bars indicate s.e.m. A difference is statistically significant if *p* ≤ 0.001 (***).

Given the severe phenotype observed, we wanted to investigate the effects of such a devastating tumor on the average lifespan, since flies did not show macroscopic alterations. For this purpose, we evaluated the average lifespan of the tumor-bearing population compared with the *insc-Gal4, ase-Gal80-GFP* control flies. The results were graphed in a Kaplan–Meier curve (Figure 2A). The graph displays that the experimental group collapses on day 14, and the difference in survival between the two genotypes is statistically significant. These individuals experienced poor motility, resulting in an inability to feed and mate, already at birth. In order to evaluate motor impairment, we performed a climbing assay, a test used in *Drosophila* to evaluate locomotor deficits. This test takes advantage from the natural tendency of the fly to climb vertically upwards, and for this reason, it is also known as the negative geotaxis assay [41]. Five groups of 100 flies each were used for this test. The flies were placed in a cylinder, and their ability to reach the top was measured. The 68% of the control progeny reached the “end line” vs. 0% of the experimental population; the tumor-bearing flies appeared immobile, remaining at the bottom of the cylinder (Figure 2B). The differences between the two genotypes were statistically significant, suggesting that the tumor-bearing population was born with a severe neuromotor deficit, since the test was performed one day after eclosion.

### 2.3. Cell Polarity Alteration in Type II NBs Causes MYC Deregulation

**Figure 3:**
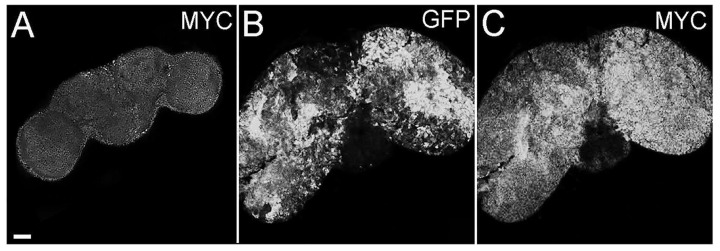
MYC staining of adult brains from *insc-GAL4*, *ase-GAL80-GFP-aPKC^CAAX-wt^* individuals. GFP marks tumor masses (**B**), and MYC protein is shown in (**C**) compared with the control *insc-GAL4*, *ase-GAL80* brain (**A**). All the images are at the same magnification (400×), and the scale bar (indicated in **A**) is 50 µm.

The proto-oncogene *MYC* is one of the most investigated genes; thanks to its central role it is defined as the “master regulator” of cellular growth and proliferation [42] and given its significant implication in tumorigenesis, it is also considered a crucial “director” of cancer growth. *MYC* paralogs are aberrantly expressed in a plethora of human cancers [43], including brain tumors [44] and GBMs [45,46]. In addition, we and others have identified *MYC* as a target of different pathways in recent studies on brain cancer carried out in the fly [32,47,48], so we investigated its expression in the presented model. Figure 3 shows that tumor cells accumulate high levels of MYC protein, contrary to control brains (Figure 3, compare C to A). As can be appreciated in the figure, MYC-positive and GFP-positive areas do not exactly overlap, and this phenomenon can be partially justified by the different persistence of MYC and GFP inside the cells; indeed, MYC has a very short half-life, while GFP is a stable, long-lasting protein. Furthermore, MYC is known to be involved in non-cell autonomous mechanisms, such as cell competition, which engages both tumor and wt cells in a highly dynamic process where MYC levels are rapidly modulated (for a review of MYC-mediated cell competition in brain cancer, see [49]). For these reasons, this analysis suggests that also in this model, MYC supports the tumorigenic process, and following molecular analyses will help confirm this piece of evidence.

### 2.4. Cell Polarity Alteration in the Type II Neuroblasts Causes Significant Changes in the Adult Brain Transcriptome

To obtain a molecular picture of the adult tumors, the samples underwent RNA-seq analysis. We performed a high-coverage (70 M–90 M read pairs/sample), high-fidelity (2 × 150 nucleotide-long reads), transcriptome-wide readout of the effects of aPKC overexpression in *Drosophila* brain samples, using Illumina RNA-Seq (see Section 4).

**Figure 4:**
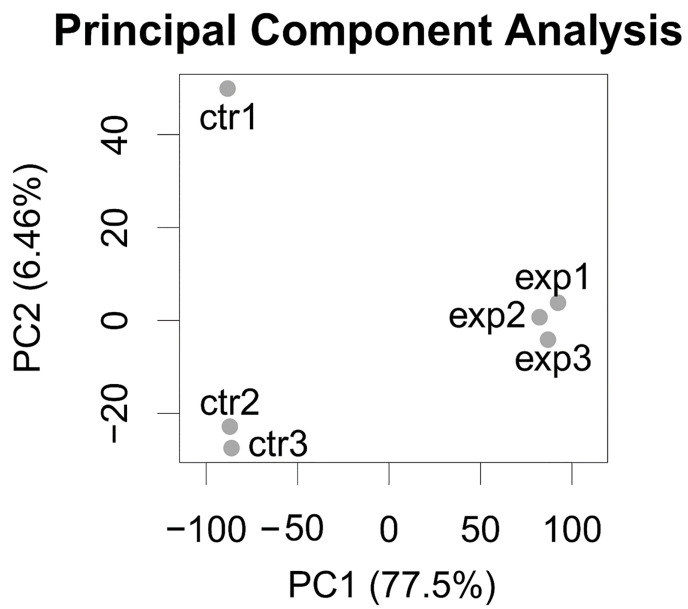
Principal Component Analysis of RNA-Seq data, showing three replicates per group. Exp: *insc-GAL4*, *ase-GAL80-aPKC^CAAX-wt^* samples. Ctr: *insc-Gal4*, *ase-Gal80-GFP* control samples. Total variance of dataset explained by components is shown as percentage in brackets on *x*-axis (Principal Component 1) and on *y*-axis (Principal Component 2).

The Principal Component Analysis performed on the gene expression profiles of the six samples (three *insc-GAL4*, *ase-GAL80-aPKC^CAAX-wt^* samples and three *insc-Gal4*, *ase-Gal80-GFP* controls) shows a good separation between the two groups and a strong aPKC transcriptome signal, running alongside the Principal Component with the highest variance (77.5% of the total dataset variance explained; Figure 4).

**Figure 5:**
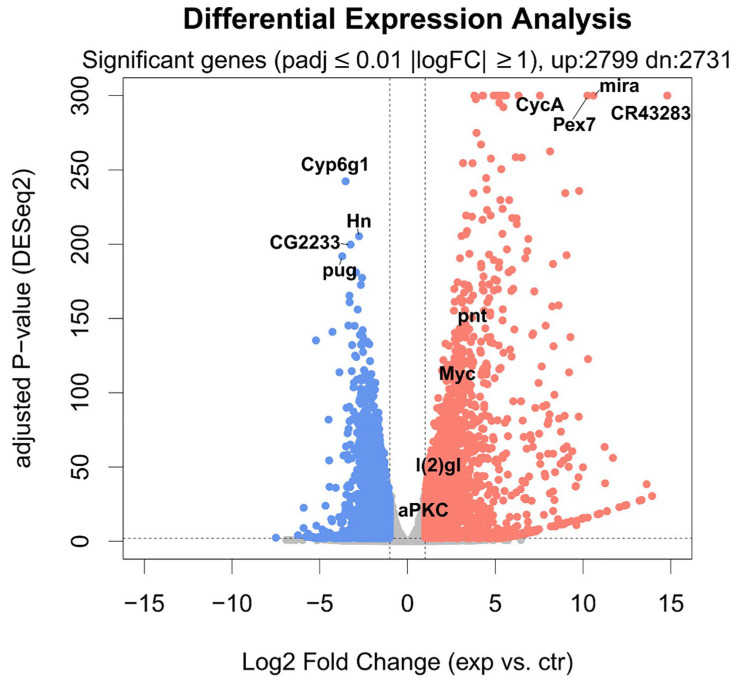
Volcano plot showing the differential expression of each gene in the aPKC vs. control contrast. *x*-Axis: Log2 Fold Change. *y*-Axis: −log10 of BH-adjusted *p*-value (calculated by DESeq2). Significant genes (adjusted *p*-value ≤ 0.01, absolute log2FC > 1) are shown in red (2799 upregulated genes) and blue (2731 downregulated genes). The 4 most upregulated and downregulated genes are shown, as well as selected genes, such as *Myc*, *pnt*, *l(2)gl*, and *aPKC*.

**Figure 6:**
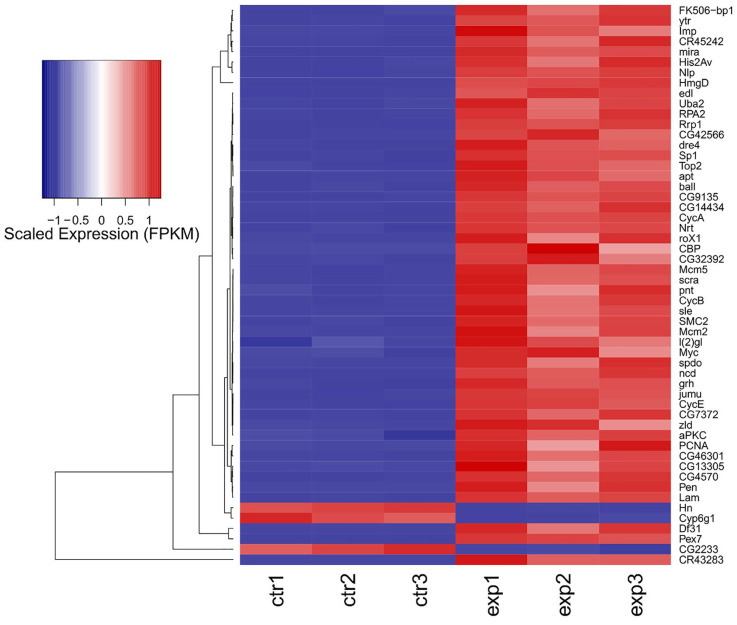
Heatmap depicting the scaled expression (FPKM: fragments per gene length in kilobases, per million mapped reads) of the 50 most differentially expressed genes in the aPKC vs. control comparison depicted in Figure 5. Individual sample values are shown as columns, and genes are shown as rows. The expression of *Myc*, *pnt*, *l(2)gl*, and *aPKC* is also depicted.

Out of 17,737 annotated *Drosophila* genes (source of the annotation: ENSEMBL), we could detect the expression of 14,745 genes. aPKC overexpression induces a major transcriptional response (Figure 5), with 5530 significantly changing genes (adjusted *p*-value ≤ 0.01 and absolute log Fold Change ≥ 1). The overexpression of aPKC was significant, and we could detect significant upregulation of neuronal development genes such as *pnt*, *Myc*, and *lgl* (Figure 5). Among the most affected transcripts, we could detect the upregulation of the stem cell-specific genes *mira* and *CR43283*, along with *CycA* and *Pex7*, and the downregulation of *Cyp6g1*, *Hn*, *CG2233*, and *pug* (Figure 6), along with strong downregulation of genes involved in the lipid catabolic process (*Lip1*, *Yip1* and *Yip2*) and the neurotransmitter transport process (*Ntl* and *CG33296*). The transcriptome-wide results, as well as the calculated best-hit human ortholog, where available (see Section 4), are provided in Table S1.

**Figure 7:**
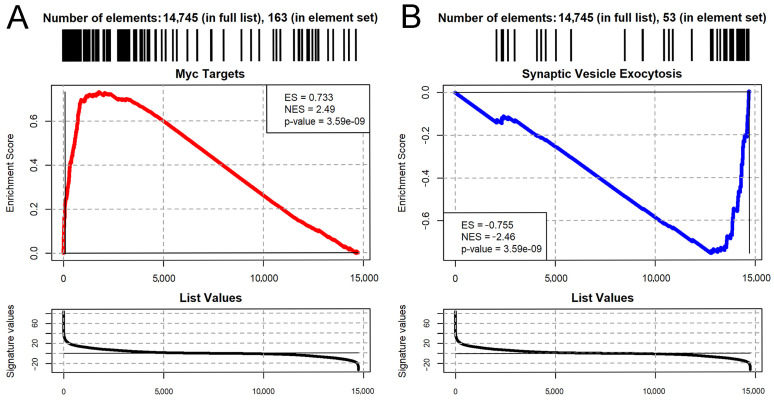
Gene Set Enrichment Analysis plots [50] showing the significant upregulation of Myc targets (**A**) and the downregulation of regulators of synaptic vesicle exocytosis (**B**). Myc targets were defined by the *Drosophila* MsigDB Hallmark pathway (“Myc targets V1”), while the pathway was defined by the Gene Ontology set “Regulation of Synaptic Vesicle Exocytosis”. ES = Enrichment Score. NES = Normalized Enrichment Score. *p*-Value is adjusted according to the Benjamini–Hochberg method.

We then performed Gene Set Enrichment Analysis (GSEA) by using the MsigDB collection of Gene Ontology, KEGG, Wiki Pathways, and Hallmark Pathways databases. A total of 1659 pathways were significantly affected by aPKC overexpression (*p*-adjusted ≤ 0.01; Table S2) In particular, we observed the general upregulation of pro-proliferative transcriptome signatures, such as cell cycle, DNA replication, protein synthesis genes, and the targets of the *Myc* oncogene (Figure 7A). At the same time, we observed the downregulation of neuronal differentiation pathways, such as ion transport genes, axon genes, and neuron projection and synaptic pathways (Figure 7B). This result is consistent with what is expected when comparing wild type adult brains, which are normally differentiated and have almost negligible neurogenesis, with tumor brains which, as demonstrated, are characterized by a large portion of cancer stem cells with a high proliferative rate, similar to those arising in humans.

### 2.5. About Half of the Most Deregulated Genes Are Known to Be Implicated in Human GMB

Starting from the 50 most deregulated genes in this model, we performed a literature review to investigate their involvement in human gliomas and GBM. The results were interesting, as for a good chunk of the genes, we found a correlation, summarized in Table 1. We identified 39 human orthologs out of the 50 genes, and of these 39, 18 are known to be implicated in GBM (about 50%). A first group of these genes, upregulated in our model, are also overexpressed in GBM cell lines or samples, although for some of them, their role in GBM progression has also been identified. A large part of these genes are more expressed in the most severe forms and correlate to a poor prognosis; for this reason, some of them, such as *TOP2A*, *PCNA*, and *VRK1*, are believed to be prognostic markers. Other subgroups of genes are involved not only in growth and proliferation but also in resistance to conventional therapies, both chemo- and radio-resistance, with APE1X being involved in both. Finally, *NOL4* and *IGF2BP2* are instead directly involved in the maintenance of Glioblastoma Stem Cells (Table 1).

**Table 1:** Human orthologs implicated in GBM. Out of the 50 most deregulated genes, we identified 39 human orthologs, among which 18 were identified involved in GBM. These genes, ordered by the log2FoldChange value, are classified into 4 categories according to their function: bad prognosis, proliferation and chemo-resistance, radio-resistance, and maintenance of GSCs. For each gene, the reference is indicated in the table.

Fly Gene	Human Gene	Implication in GMB	Ref.
*CycA*	*CCNA2*	Bad Prognosis	[51]
*Lam*	*LMNB2*		[52]
*Pen*	*KPNA2*		[53]
*Top2*	*TOP2A*		[54]
*PCNA*	*PCNA*		[55]
*ball*	*VRK1*		[56]
*His2Av*	*H2AFV*		[57]
*Mcm2*	*MCM2*		[58]
*Mcm5*	*MCM5*		[58]
*HmgD*	*HMGB2*	Chemo-Resistance	[59]
*ncd*	*KIFC1*		[60]
*CycE*	*CCNE1*		[61]
*Rrp1*	*APEX1*		[62]
*Rrp1*	*APEX1*	Radio-Resistance	[63]
*CG9135*	*RCC2*		[64]
*CG42566*	*LITAF*		[65]
*RPA2*	*RPA2*		[66]
*CG46301*	*NOL4*	Maintenance of GSCs	[67]
*Imp*	*IGF2BP2*		[68]

## 3. Discussion

GBM is the most common and lethal adult brain tumor. Over the years, the role of GSCs appears to be increasingly important in this tumorigenic process, and given their crucial role in the initiation, growth, and recurrence of GBM, they represent an interesting therapeutic target [69]. The origin of GSCs is still strongly debated, even if it appears increasingly likely that they derive mainly from the reprogramming of normal neural progenitors; therefore, it is important to decipher the crucial mechanisms driving their transformation. With this in mind, our aim was to build a neurogenic brain tumor model, focusing our attention on the role of cell polarity in this neoplastic process.

In our previous work, cell polarity was altered by the deregulation of the PTEN/aPKC/Lgl axis using the Optix promoter, which is expressed in a well-defined compartment of the OPC neuroepithelium, in one type I NB and in four of the eight type II NBs, as well as in most of the INPs, GMCs, and neurons of these lineages [70]. The evidence that aPKC deregulation in the Optix region leads to the onset of tumors that originate in the central brain and are characterized by type II-like NBs suggests that these NBs may represent the cells of origin of these malignant masses. To confirm this, we narrowed the target area by inducing aPKC activation in the eight type II NBs. The resulting masses were mainly composed of immature progenitors, identified by the expression of Miranda. Those progenitor cells, instead of exiting the cell cycle and differentiate into glia or neurons, as normally happens, persisted in the adult stage. Furthermore, these neuroblasts continued to proliferate in the adult individual, as demonstrated by the positivity for PH3, a specific marker of mitosis (Figure 1). These cells are normally not observable in the adult brain of *Drosophila*; in fact, even if adult neurogenesis is now consolidated, it has not yet been possible to identify adult neural stem cells, nor has it been possible to identify cells that divide in a normal brain [40]. These tumors were highly invasive; they almost completely filled the organ, altering its structure and leading the animals to untimely death. Considering the significant impairment of the central nervous system and given that type II NBs give rise, among others, to structures implicated in orientation and the directional control of movements [71], we wanted to verify the tumor impact on the locomotor abilities of bearers. We performed a climbing assay, which confirmed that these flies have neuromotor deficits, remaining practically immobile with the consequent inability to mate and eat. Several types of human brain tumors, including GBM, present the upregulation of the transcriptional regulators belonging to the MYC family [72]. MYC proteins are expressed in a region-specific manner in different stages of the developing brain and regulate neural development in mammals and non-mammalian vertebrates [73]. The most part of information on MYC functions in neural development was collected in invertebrates such as *Drosophila*, with which mammals share the organization and cell division control mechanisms of neural stem cells, thanks to the presence of specific, conserved determinants. In *Drosophila*, MYC is highly expressed in neuroblasts and less in late progenitors [74]; in the formers, MYC levels are high because it promotes self-renewal while inhibiting differentiation, so as development proceeds, the levels of MYC are gradually lowered, which allows the cells to exit cell cycle and differentiate. Therefore, MYC levels are strictly regulated to finely control the balance between proliferation and differentiation [74]. Beyond the physiologic role of MYC, its involvement in the maintenance of GSCs is also increasingly evident, both in *Drosophila* and humans [47,75]. Given the well-known function of MYC in the maintenance of both normal and tumor neural stem cells, we decided to investigate MYC protein levels in our model through an immunofluorescence analysis. As can be seen in Figure 3, the tumor areas (GFP-positive) show widespread accumulation of the MYC protein (in red), suggesting that cell polarity disruption alters the mechanisms necessary to regulate MYC, whose persistence, in turn, prevents NB differentiation pathways, causing the expansion of immature progenitors in the adult organ. MYC overexpression in human tumors may depend on various mechanisms: transcriptional regulation, gene amplification, and translational and post-translational regulation (reviewed in [76]). One of the most common causes of MYC deregulation is protein stabilization. MYC is an unstable protein, with a half-life of about 15–30 min. Generally, MYC degradation occurs following the double phosphorylation of S62 by ERK [77] and T58 by GSK-3β [78]. GSK-3β is inhibited by aPKC phosphorylation [79], so MYC accumulation in our model may be due to aPKC sustained activation. This brain tumor model initiated from type II NBs summarizes many of the typical traits of human brain cancer and, in particular, of GBM. Starting from this evidence, we decided to carry out an RNA-seq analysis with the aim of identifying a specific signature of cell polarity disruption. As can be seen in the volcano plot (Figure 5), the alteration in cell polarity in the type II neuroblasts causes important changes in gene expression in the adult brain, with 5.530 significantly changing genes. For each *Drosophila* gene, we identified the human ortholog, where possible. Among the significantly upregulated transcripts, we observed several genes of interest, some of which were also upregulated at the protein level, as confirmed by the IF assays. One of these is *aPKC*, which represents an internal control of the system, but we also found the transcriptional upregulation of *Miranda, PntP1*, and *MYC*. Given the large number of deregulated genes (up and down), we decided to focus our attention on the 50 most deregulated genes, represented in the heatmap in Figure 6, by interrogating the literature in search of a possible involvement of their human orthologs in gliomagenesis. We identified 39 human orthologs, and among these, 18 were involved in GBM, playing different roles, as summarized in Table 1. Different genes upregulated in our model were also found to be upregulated in different cell lines and/or in GBM samples compared with normal brain tissues. Among these, *LMNB2*, encoding nuclear type B2 Lamin, is significantly upregulated in glioma, and it has been observed that the high expression of the laminin family members in glioma cells is associated with rapid progression of the disease, suggesting a direct role in gliomagenesis [52]. Also, *H2AFV*, one of the histone 2A family members, is upregulated, in particular, in proneural-type GBM [57]. Others act as prognostic markers. For example, KPNA2 (Karyopherin Subunit Alpha 2), also known as importin α-1, is considered a novel biomarker of recurrence and malignant progression for astrocytic gliomas [53]. The upregulation of TOP2A (DNA Topoisomerase II Alpha) is also a prognostic biomarker in patients with glioma [54]. A direct correlation is also observed between the levels of expression of PCNA (Proliferating Cell Nuclear Antigen) and the histological grade of CNS tumors; PCNA, in fact, is used to evaluate malignancy in CNS tumors and estimate the probability of relapses [53]. The levels of VRK1 (Vaccinia-related kinase 1) also show a clinical significance in human gliomas, and high levels of VRK1 are indeed associated with a poor prognosis [56]. MCM2 and MCM5 are two members of the Minichromosome Maintenance (MCM) family; the entire family of MCMs is promising in glioma prognosis and diagnosis [58]. CCNA2 or Cyclin A, a member of the cyclin family, is not expressed in tumor cells but is a key gene of GASCs (Glioblastoma-Associated Stromal Cells), promoting glioblastoma growth and regulating the cell cycle [51]. Some products of these genes are instead involved in chemo-resistance mechanisms. HMGB2 (High-Mobility Group Box 2), for example, is associated to a poor prognosis, promoting invasion and chemo-resistance to temozolomide (TMZ), the gold standard in GBM chemotherapy [59]. KIFC1, also known as HSET, enhances TMZ resistance by promoting DNA repair instead [63]. CCNE1, Cyclin-E1, is upregulated in TMZ-resistant cells [61]. Finally, APEX1 (DNA- (apurinic or apyrimidinic site) endonuclease) is implicated not only in TMZ chemotherapy [60] but also in radio-resistance, regulating DNA damage repair [63]. There are other genes directly involved in radio-resistance, such as *RCC2* (Regulator of Chromosome Condensation 2), also known as *TD60*, which advances proliferation and radio-resistance in GBM [64]. The transcription factor LITAF promotes radiosensitivity in gliomas. Additionally, RPA2 (Replication Protein A2), governs the radio-resistance of GSCs [66]. Ultimately, two of these genes have a direct role in Glioblastoma Stem Cells: *NOL4* (nucleolar protein 4) was identified as 1 of 20 genes expressed in GSCs but not in NSCs and, for this, as new possible therapeutic target [67]. *IGFBP2* (Insulin-like growth factor 2 mRNA-binding protein 2), also known as *IMP2*, is an mRNA-binding protein that regulates a large number of cellular processes, and it is implicated in glioma progression [80] by maintaining GSCs [68,81].

As we can see, there are several genes that are found to be deregulated in our invertebrate model that are known to be involved, with different functions, in human GBM. At this point, we wondered if some of these genes performed their function via MYC, given our interest in the role of this oncoprotein in the gliomagenic process. For example, the action mechanism of KPNA2, a prognostic marker, in tumorigenesis and the progression of glioma occurs in part by regulating metabolism [82]. Glioma cells, like other highly proliferating tumor cells, reprogram their metabolism, performing glycolysis even in aerobic conditions, a phenomenon known as the “Warburg effect” [83]. One of the genes responsible for the metabolic reprogramming of cancer cells is MYC, which, among other functions, enhances glycolysis [84]. Also in this case, KPNA2 reprograms the metabolism of GBM cells, regulating *c-myc* [82]. Among other genes, however, *IMP2* controls GBM progression, regulating OXPHOS, on which the survival of GSCs depends. In *Drosophila*, *IMP* has been observed to regulate the size and proliferation rate of NBs by stabilizing *MYC* mRNA, thus enhancing self-renewal [85]. IGF2BPs are overexpressed in metastatic cancers, and their expression is associated with that of important oncogenes, such as MYC. There is evidence that in different cancers, IGF2BPs increase MYC expression, stabilizing the transcript and preventing its degradation [86]. It would thus be interesting to observe whether this mechanism also occurs in GBM. The evident role of MYC in the gliomagenic process is also suggested by the pathway enrichment analysis we carried out. The upregulated genes are involved in biological processes related to proliferation and, in particular, we observed the upregulation of MYC targets and the downregulation of neuronal differentiation genes (Figure 7). The alteration in cell polarity in type II NBs probably involves a shift in their division from asymmetric to symmetric, causing the accumulation of NBs that no longer continue to undergo differentiation, ultimately leading to the formation of an adult tumor. This would explain not only the upregulation of pro-proliferative genes in our tumors but also the downregulation of genes specific to mature neural cells. Type II NBs, thanks to their particular lineage, generate a very high number of neurons and mature glia [36]; therefore, their exit from the differentiation pathways would consequently lead to the loss of numerous adult cells. Summarizing, we observed how the alteration in cell polarity in type II neuroblasts, a little subpopulation of the *Drosophila* neural stem cells, causes the formation of adult tumor masses composed of mitotic cells which continue to proliferate even in the adult individual. These tumors have a significant impact on the life of the affected individuals, causing neuro-motor damage and leading to premature death. The tumor masses also express high levels of MYC oncoprotein, which is known to play an important role both in CNS development and numerous types of tumors, including brain cancers. We investigated how the transcriptome of these tumors varied compared with normal adult brains, observing that there is general upregulation of the genes involved in proliferation, including MYC and its targets, and downregulation of genes related to neural maturation. Finally, we focused on the 50 most deregulated genes and investigated their hypothetical, known role in GBM, which we found for about half of them. Several upregulated genes in our model are also upregulated in human GBM, functioning as prognostic markers, while others are involved in chemo- and radio-resistance or in the maintenance of GBM cancer stem cells. Our analysis identified type II neuroblasts (NBs) as extremely sensitive to aPKC-induced cell polarity alterations. Type II NBs are a subpopulation of *Drosophila* stem cells displaying a lineage similar to that of mammalian neural stem cells, involving transient amplifying cells expanding the pool of progenitors. Therefore, this neurogenic brain tumor model recapitulates the essential phenotypic and molecular characteristics of mammalian brain tumors.

In light of these findings, we conclude that our system is highly suitable to deepen the genetic and molecular knowledge of human gliomas and GBM. Furthermore, given the recent subdivision of GBM into different sub-types, which are likely to originate from different progenitors [87], our model meets the need to broaden the offer of animal models that can help address different aspects of this complex disease.

## 4. Materials and Methods

*Fly Stocks.* The following fly stocks were used in the study: *w*; *UAS-*aPKC*^CAAX-wt^-w*; *insc-Gal4*, *UAS-EGFP/CyO*; *ase-Gal80/TM6b-w*; *UAS-EGFP/CyO*. Fly lines were from the Bloomington Stock Center. *Drosophila* stocks were raised at 25 °C, and experimental crosses were kept at 29 °C.

*Immunohistochemistry.* Adult brains were dissected in PBS, fixed in 3.7% formaldehyde in PBS for 30 min, permeabilized in 0.5% Triton in PBS for 2 h, and stained with standard protocol. The following primary antibodies were used: mouse anti-Repo (1:100; Developmental Studies Hybridoma Bank), anti-Elav (1:100; Developmental Studies Hybridoma Bank); rabbit anti-Mira (1:200; C.Q. Doe); rabbit anti-PH3 (1:200; Ser10 Upstate Biotechnology, Boston, MA, USA); mouse anti-MYC (1:5; P. Bellosta); mouse anti-dIAP1 (1: 200; B.A. Hay). Secondary antibodies were Alexa Fluor 555 goat anti-mouse and anti-rabbit (Invitrogen Corporation, Carlsbad, CA, USA) and DyLight 649 goat anti-mouse and anti-rabbit (Jackson Immuno Research Laboratories, West Grove, PA, USA). Fluorescent images were acquired with a Leica TCS SP2 confocal microscope, and images were processed as a whole by using Adobe Photoshop software. For immunofluorescence analysis, the figures represent the average phenotype across 10–20 samples analyzed, and all the images represent a 1μ single z stack.

*Lifespan.* The lifespan assay was performed by keeping the adult flies in vials (20 flies/vial) and rearing them at 29 °C. Surviving flies were transferred on fresh medium and counted every two days until 28 days after eclosion. At least 100 flies were tested for each genotype, and only female flies were used.

*Climbing assay.* Flies were collected at birth and raised in batches of 20. Flies were transferred into a 50 mL glass graduated cylinder and allowed 10 min to acclimate. The climbing ability of flies was quantified as the number of animals that reached the arbitrary height of 7.5 cm in 10 s. This step was repeated 10 times, with one minute of rest between steps. At least 100 flies (five biological replicates of 20 flies) were tested for each genotype, and only female flies were used.

*Statistical Analysis*. For the analysis of average survival, Kaplan–Meier curves were generated, and differences between the curves were evaluated by using the Χ-square test. The long-rank test (Mantel–Cox) was used. For the climbing assay, a two-tailed Student’s *t*-test was performed. The *p*-values were *p* ≤ 0.05 = *, *p* ≤ 0.01 = **, and *p* ≤ 0.001 = ***. All data were statistically analyzed, and all graphs were created with GraphPad Prism 5.

*RNA extraction*. RNA was obtained from the heads of 1-day-old adult flies. A total of 30 heads were collected for each sample, for a total of 6 samples (3 replicates for experimental samples and 3 replicates for control samples). The *Drosophila* heads, collected in a vial containing 1 ml of TRI Reagent^®^ (Sigma-Aldrich, Burlington, MA, USA), were homogenized with a disposable polypropylene pestle and centrifuged at 12,000× *g* at 4 °C to eliminate debris. The supernatant was transferred in new vials containing 200 μL of chloroform, vortexed, incubated for 10 min at RT, and centrifuged for 15 min at 12,000× *g* at 4 °C. RNA-containing aqueous phase was isopropanol-precipitated. The supernatant was removed, and the pellet was washed three times with 1 mL of 75% EtOH and centrifuged at 7500× *g* for 5 min at 4 °C. After supernatant removal, the pellet was resuspended in 30 μL of nuclease-free water at 55 °C for 10 min. The total RNA quantity was obtained with a NanoDrop™ 2000/2000 c Spectrophotometer. Total RNA was stocked at −80 °C before being shipped on dry ice to the Genewiz^®^ facility (GENEWIZ Germany GmbH, Leipzig, Germany) for sequencing.

*Illumina RNA sequencing and data analyses.* RNAs were processed by using the Illumina TruSeq kit and sequenced via Illumina NextSeq550, providing paired reverse-stranded sequencing fragments (read pairs with 2 × 150-nucleotide-long reads). The number of fragments was in the range of 70 M–90 M per sample (ctr1: 73.4 M; ctr2: 80.1 M; ctr3: 89.5 M; exp1: 75.8 M; exp2: 79.2 M; exp3: 90.5 M). Read pairs were trimmed by using fastp [88] and aligned over the *Drosophila* dm6 genome obtained from Flybase [89], using Hisat2 aligner version 2.1.0 [90]. Alignment files (SAMs) were compressed and sorted via samtools suite version 1.10 by using htslib version 1.10.2-3 [91]. Gene expression counting was performed by using the featureCounts command from the subread package with 12 threads and default parameters [92]. Genome annotation for gene expression was obtained from ENSEMBL, using *Drosophila melanogaster* annotation BDGP6.95 in GTF format [93]. Differential expression analysis was performed by using DESeq2 [94] on a simple design (3 overexpressors vs. 3 controls), and the *p*-value was adjusted according to the Benjamini–Hochberg method [95]. R custom scripts (R version 4.1.1) and the corto package [96] were used for common bioinformatics tasks such as conversion from gene counts to FPKMs, Principal Component Analysis (PCA), volcano plots, heatmaps, and Gene Set Enrichment Analysis (GSEA) [50]. The MsigDB database including the KEGG, Wiki Pathways, Gene Ontology, and Hallmark collections was used for gene pathway definition, using R package msigdbr version 7.4.1 [97]. The assignment of the most likely human ortholog for each *Drosophila* gene was performed by using data from DIOPT (DRSC Integrative Ortholog Prediction Tool [98]); when multiple human genes were equally scored as candidate orthologs for a *Drosophila* gene, we selected the most expressed gene (by FPKM expression) in the TCGA (The Cancer Genome Atlas) Glioblastoma dataset. All computational steps were performed on a workstation equipped with an AMD Ryzen 9 5900X 12-Core Processor, 64 GB of DDR4 RAM by Corsair at 3600 MHz, a Samsung MZ-V8P2T0B 980 PRO NVMe 2 TB hard drive, and an MSI MAG B550 Tomahawk motherboard, running on a Xubuntu 20.04 LTS Operating System.

## Data Availability

The data produced are available as Supplementary Materials.

## Supplementary Materials

The following supporting information can be downloaded at: https://www.mdpi.com/article/10.3390/ijms26115115/s1.
